# Correspondence

**Published:** 1984-04

**Authors:** C. J. Burns-Cox

**Affiliations:** Frenchay Hospital


### Correspondence

Sir

When Mr. Eyre-Brook left Malawi he needed to find a replacement. He contacted the Bureau for Overseas Medical Service and within 2 days had the names and addresses of 2 consultant orthopaedic surgeons who had previously said they were keen to work for a few months overseas. One of them had a bad back but the other immediately agreed to go!

I am writing this letter to publicise the Bureau for Overseas Medical Service, Africa Centre, 38 King Street London WC2E 8JT, tel. 01 836 5883. Doctors or para-medical workers keen to do a stint in the 3rd world please write and register with this organisation who will then let you know when a suitable vacancy arises. There are usually around 50 medical and many para-medical vacancies at any one time.

C. J. BURNS-COX,
Frenchay Hospital

